# The regulation of snail: on the ubiquitin edge

**Published:** 2017-07-03

**Authors:** Qian Yu, Binhua P. Zhou, Yadi Wu

**Affiliations:** 1 Pharmacology and Nutritional Sciences, the University of Kentucky, College of Medicine, Lexington, KY 40506, USA; 2 Molecular and Cellular Biochemistry, the University of Kentucky, College of Medicine, Lexington, KY 40506, USA; 3 Markey Cancer Center, the University of Kentucky, College of Medicine, Lexington, KY 40506, USA

**Keywords:** metastasis, EMT, Snail, Dub3, ubiquitination

## Abstract

Metastasis accounts for a majority of cancer death. One key feature during metastasis is epithelial-mesenchymal transition (EMT), which is regulated by transcription factors such as Snail and Twist. In non-malignant cells, Snail has a short half-life and is degraded via ubiquitination, but its stability is increased in cancer cell. However, the mechanism by which Snail escapes ubiquitination and degradation remains unknown. Recently, we found that Dub3 is a deubiquinase of Snail. Most importantly, we determined that Dub3 responded to extracellular signals such as IL-6, and that the resultant signaling prevented Snail degradation, and promoted cancer growth, invasion, and migration. In this highlight, we present a concise picture of how the transcription factor Snail is regulated by ubiquitination in cancer cells, the role of Dub3 in this process, and its potential use as a treatment target.

## The ubiquitination of Snail

Metastasis accounts for 90% of cancer deaths ^[1]^. In order to establish new growth at a distant location, cancer cells undergo invasion, intravasation, extravasation, and metastatic colonization ^[2–4]^. During this process, EMT is necessary for cancer cells to lose their original intercellular adhesion and initially adopt an increased motility ^[5, 6]^. Similar to the establishment of gestation layers by embryological cells, cancer cells undergoing EMT re-overexpress transcription factors such as Slug, Twist, and Snail, which repress proteins such as cadherin, claudin, integrin, cytokine, as well as others ^[5, 7]^. Among these molecular triggers, Snail, a zinc finger protein family member, has been widely acknowledged for its role in EMT ^[5, 8–10]^. Clinically, Snail expression is associated with resistance to chemotherapy, decreased survival, high recurrence rates and a poor prognosis ^[11–13]^. In accordance with its profound role in development, Snail has a short half-life and is tightly regulated by extracellular signaling, induced phosphorylation and degradation through ubiquitination ^[14]^.

**Figure 1:**
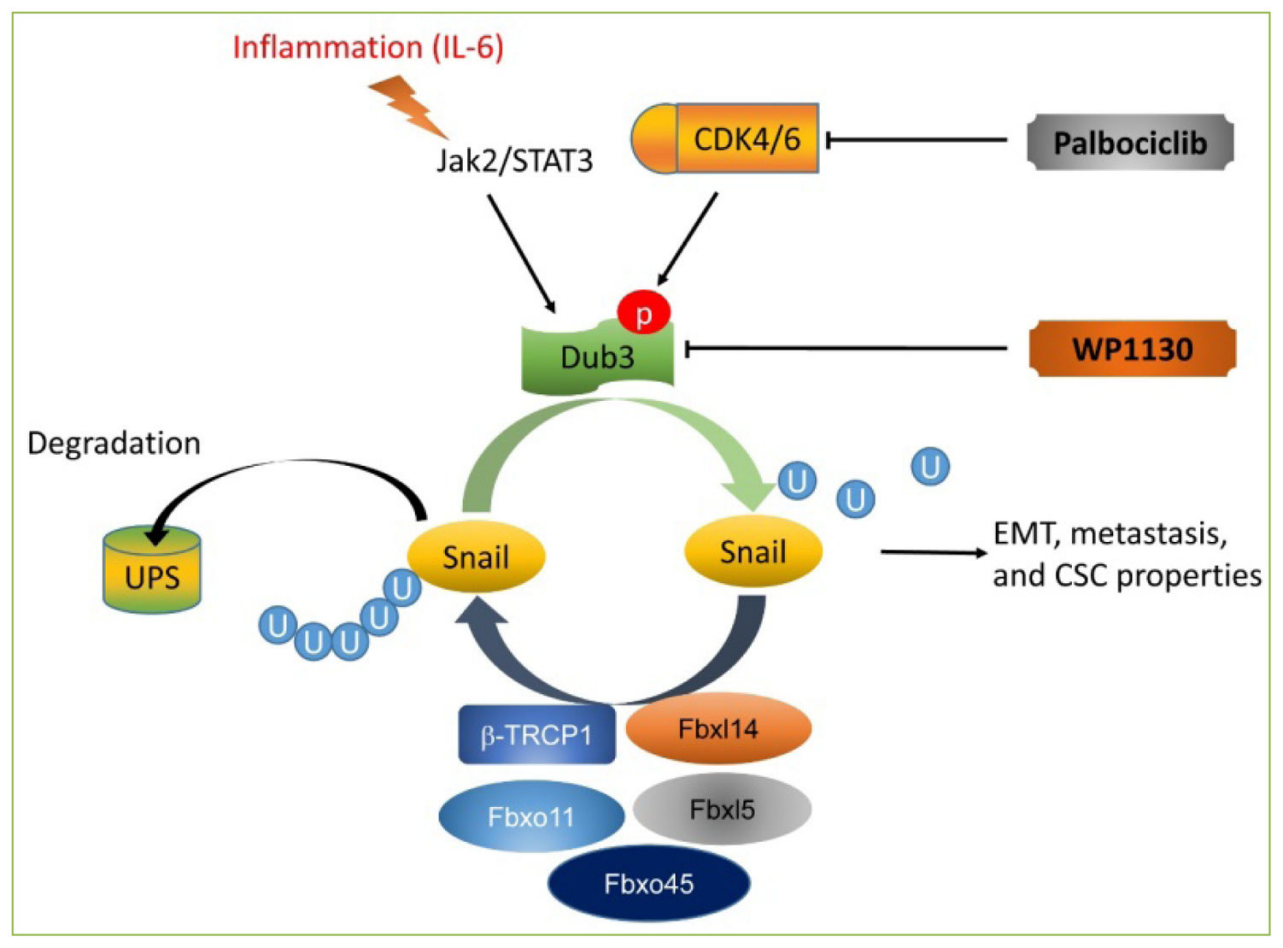
The ubiquitination regulation of Snail Snail is degraded by several E3 ligase including β-TRCP1, Fbxl14, Fbxl5, FBXO11 and Fbxo45. By contrast, Dub3 deubiquitinates Snail and prevents snail degradation through two axis: (1) the IL-6/Dub3/Snail axis: the extracellular IL-6 signal leads to increased Dub3 activity; (2) CDK4/6/Dub3/Snail axis: CDK4/6 phosphorylates Dub3 at Ser41. Dub3 is responsible for EMT, cancer stem cell like traits, and invasiveness through Snail stabilization.

The ubiquitination proteasome pathway is one approach utilized by cells to degrade targeted protein in eukaryotic cell ^[5]^. During this process, ubiquitin, a 76 aminoacid peptide, initially forms a thio-ester bond with the ubiquitin activating enzyme (E1) and then is transferred to a ubiquitin conjugating enzyme (E2). Subsequently, a ubiquitin ligating enzyme (E3) catalyzes the formation of a covalent bond between ubiquitin and a lysine residue on the target protein, which then becomes susceptible to proteasomal degradation ^[15]^. Snail’s ubiquitination and degradation are controlled by several F-box ligases, including β-TRCP1/Fbxw1, Fbxl14, Fbxl5, Fbxo11, and Fbxo45 ^[16–22]^ (Figure 1). β-TRCP1/Fbxw1 is one of the best-characterized Snail E3 ligase. To initiate the even, phosphorylation of Snail by GSK3β on residues S104 and S107 promotes Snail localization to the cytoplasm; additional phosphorylation events by GSK3β on the S96 and S100 residues generate the β-TRCP1 phosphodegron ^[17]^. Fbxl14, a cytoplasmic F-box protein, shares the same lysine residues as β-TRCP1 in the Snail N-terminal domain: K98, K137 and K146 and through these associations ligates ubiquitin to Snail ^[21]^. Through global shRNA screening of F-box proteins, Fbxl5 was identified as targeting Snail. Fbxl5 is localized predominantly in the nucleus. Interestingly, Fbxl5 interacts with the Snail C-terminus, the region involved in binding to the E-box sequence while it polyubiquitinates Snail on its N-terminal residues K85 and K146 as well as on its C-terminal residue K234 ^[18]^. In addition, Fbxl5-induced Snail down-regulation is impaired by post-translational modifications that prevent nuclear export, such as Lats2 phosphorylation ^[23]^. Fbxo11 is another ubiquitin ligase that targets the SNAG domain of Snail ^[19,24]^. Whether Snail degradation by Fbxo11 is dependent on S11 phosphorylation by the PKD1 is controversial and needs confirmation. The role of PKD1–induced Snail phosphorylation is complex since PKD1 can either inhibit or enhance Snail transcriptional repression ^[25, 26]^. Fbxo45 is an atypical F-box protein induced by estrogen ^[27]^. Fbxo45 interacts with Snail through its F-box domain. Surprisingly, several of these E3 ligases interacts as well with Slug, Zeb1 and Twist such as β-TRCP1, Fbxl14 and Fbxo45 while only Fbxl5 specifically interacts with Snail. Previously, we found that the expression of Snail can be stabilized by NF-κB at both transcriptional and post-translational levels, to downregulate the expression of E-cadherin and facilitate EMT ^[22, 28]^.

## The deubiquitination of Snail

One way to counteract this E3 ligase-mediated protein degradation is through deubiquitinase, which removes the ubiquitin moieties from target proteins and increases their stability ^[29]^. Deubiquitinase enzymes (DUBs) are classified as UCH, USP, JAMM, OTU, and Ataxin-3/Josephin group. Growing evidence shows that DUBs are essential for the regulation of many cellular functions including transcription, DNA repair, apoptosis and cell cycle progression ^[30]^. Dub3 belongs to the USP group. Whereas Dub1 and Dub2 were identified in mice, Dub3 is their human counterpart ^[31]^. This family of growth-regulatory deubiquitinating enzymes are immediate-early genes and are induced rapidly and transiently in response to cytokine stimuli ^[32]^. Within last few years, the role of Dub3 has emerged as an important deubiquitinase in DNA repair, cell proliferation, transcriptional factor regulation and tumor metastasis. For example, by deubiquitinating histones H2A and H2B, Dub3 impedes the recruitment of DNA repair factors such as 53BP1 and BRCA1 and cripple the DNA damage response that occurs with genotoxic stress ^[33]^. By decreasing the turnover rate of cdc25A phosphatase, Dub3 facilitates the G1/S transition and promote cancer cell proliferation ^[34,35]^. Dub3 also regulate YAP/TAZ activity by controlling the stability of the E3 ligase ITCH, the LATS kinases and the AMOT family proteins ^[36]^. Using siRNA library screen targeting the 99 known or putative DUBs, we identified Dub3 is a deubiquitinase of Snail. Dub3 stabilizes Snail via deubiqutination, downregulating E-cadherin expression and promoting EMT ^[37, 38]^. The interaction of Snail with Dub3 is mediated at the N-terminal region of Snail, which holds the SNAG domain and is the most common binding site for E3 ^[38]^. Expression of Dub3 blocked Snail degradation mediated by the E3 ligases Fbxl14 and Fbxw1/β-TRCP1 ^[38]^. Functionally, we found that the expression of Dub3 in breast cancer cell lines not only downregulates markers of luminal type of breast cancer, but also upregulates molecules associated with BLBC ^[38]^. Knockdown of Dub3 inhibits migration, invasion and cancer stem cell (CSC)-like characteristics in cells by downregulating of Snail. By contrast, ectopic expression of Dub3 induces EMT through an upregulation of Snail. Most importantly, we found that depletion of Dub3 not only dampened spontaneous lung metastasis but also inhibited tumor recurrence. Clinically, Dub3 overexpression predicts a shortened relapse-free survival and higher probability of distant metastasis. These results strongly suggest that Dub3 can be a therapeutic target.

IL-6, a major cytokine present in the tumor microenvironment, can induce EMT and promote metastasis through the STAT3 signaling pathway in breast cancer, head and neck cancer and pancreatic cancer ^[39]^. Increased IL-6 level predicts tumor recurrence, a poor response to chemotherapy, poor survival, and tumor metastasis ^[40]^. IL-6 is also identified as a major cytokine secreted by BLBC cells and is essential for the CSC-like characteristic of BLBC ^[41]^. Interestingly, Dub3 activity and expression, at both the mRNA and protein levels, can be induced by IL-6 through the JAK/STAT3 signaling pathway ^[31, 38, 39]^. Consistent with this, we also found that IL-6 rapidly induces Dub3 expression, exerting a maximum effect at 2hr ^[38]^. Dub3-knockdown not only reduced the endogenous level of Snail but also blocked IL-6-induced Snail stabilization and invasion. Thus, DUB3 could be a missing bridge between the extracellular signaling in the tumor environment and intracellular changes in genomic expression, and explains how tumor-promoting inflammation can facilitate cancer metastasis. These data delineate a key role by Dub3 in connecting extracellular inflammatory signals to invasion, CSC-like traits, and drug resistance.

Considering the crucial role of Dub3 in triggering EMT and metastasis, we screened several Dub3 inhibitors and found that WP1130 could bind to the catalytic site of Dub3. *In vitro*, WP1130 inhibited cell migration, invasion, and mammosphere formation, whereas *in vivo*, tumor growth decreased significantly after treatment ^[38]^. By blocking the interaction of Dub3 and Snail, WP1130 prevents the deubiquitination of Snail, thereby blocking cancer invasion, migration, and the establishment of CSC-like properties. Concurrently, palbociclib, a specific inhibitor of CDK4/6, was reported to decrease Snail stability in a Dub3-dependent manner ^[37]^. CDK4/6 phosphorylates Dub3 at Ser41, a crucial site for Dub3 activity in the regulation of Snail’s stability ^[37]^. By acting on the CDK4/6-Dub3-Snail axis, palbociclib reduced cancer cell migration and metastasis *in vitro* and *in vivo*. In both cases, the ultimate target was the Dub3-Snail interaction, suggesting critical role of Dub3-Snail axis in cancer progression and metastasis (Figure 1).

## Conclusion

Snail has been well acknowledged for its importance in mediating EMT and tumor metastasis. Its stability is crucial for cancer progression and metastasis. We have identified Dub3 as the key regulator of Snail and the missing link between extracellular signaling from sources such as IL-6 in tumor environment, and the gene expression changes in cells that promote cancer. In addition, we exploited Dub3’s value as a therapeutic target. WP1130 reduces cancer migration and metastasis by inhibiting Dub3’s catalytic activity and thereby restores the ubiquitination-mediated degradation of Snail. Based on Dub3’s dual role in regulating tumor growth and metastasis, future clinical and pharmaceutical investigations could be directed towards this specific interaction of Dub3-Snail. Last but not least, while our study indicated that Dub3 could counteract the function of β-TRCP1 and Fbxl14 in regulating the degradation of Snail, both β-TRCP1 and Fbxl14 could also influence the stability of Slug, ZEB1 and Twist. Future research could explore Dub3’s role in regulating these transcription factors ^[21,42,43]^.

## Abbreviations

PKD1: Protein Kinase D 1
GSK3β: Glycogen Synthase Kinase3
FBXW1: F-box/WD repeat-containing protein 1
Fbxl: F-box and leucine-rich repeat protein
Fbxo: F-boxes other
UCH: ubiquitin C-terminal hydrolase
USP: ubiquitin-specific processing proteases
JAMM: Jab1/Pad1/MPN domain containing metallo-enzymes
OUT: Otu domain ubiquitin-aldehyde binding proteins
Ataxin-3/Josephin: Ataxin-3/Josephin domain containing proteins
53BP1: p53 binding protein 1
BRCA1: Breast Cancer gene 1
cdc25a: Cell Division Cycle 25 Homolog A
YAP: Yes Associated Protein 1
TAZ: Tafazzin
ITCH: Itchy E3 Ubiquitin Protein Ligase
LATS: Large Tumor Suppressor Kinase
AMOT: Angiomotin
IL-6: Interleukin-6
JAK: Janus Kinase
STAT3: Signal Transducer And Activator Of Transcription 3
CDK: Cyclin Dependent Kinase
BLBC: basal-like breast cancer
